# Novel point-of-care biomarkers of the dry anophthalmic socket syndrome: tear film osmolarity and matrix metalloproteinase 9 immunoassay

**DOI:** 10.1007/s00417-022-05895-0

**Published:** 2022-11-11

**Authors:** Alexander C. Rokohl, Katharina Wall, Marc Trester, Philomena A. Wawer Matos, Yongwei Guo, Werner Adler, Keith R. Pine, Ludwig M. Heindl

**Affiliations:** 1 Department of Ophthalmology, University of Cologne, Faculty of Medicine and University Hospital of Cologne, Kerpener Straße 62, 50937 Cologne, Germany; 2 Center for Integrated Oncology (CIO), Aachen-Bonn-Cologne-Düsseldorf, Cologne, Germany; 3 Trester-Institute for Ocular Prosthetics and Artificial Eyes, Cologne, Germany; 4 Eye Center, Second Affiliated Hospital, School of Medicine, Zhejiang University, Hangzhou, China; 5 Department of Biometry and Epidemiology, Friedrich-Alexander University Erlangen-Nuremberg, Erlangen, Germany; 6 School of Optometry and Vision Science, University of Auckland, Auckland, New Zealand

**Keywords:** Dry anophthalmic socket syndrome, Tear film osmolarity, Matrix metalloproteinase 9, Enucleation, Evisceration, Conjunctival inflammation, Anophthalmic socket surface inflammation

## Abstract

**Purpose:**

To compare tear film osmolarity (TFO) values and matrix metalloproteinase 9 (MMP-9) levels between anophthalmic sockets and healthy fellow eyes and to assess the use of the MMP-9 and TFO as objective biomarkers for the dry anophthalmic socket syndrome (DASS).

**Methods:**

In this prospective single-center study, the anophthalmic sockets and healthy fellow eyes of 98 unilateral anophthalmic patients were assessed using the ocular surface disease index (OSDI) questionnaire, InflammaDry® MMP-9 point-of-care immunoassay, TFO with TearLab™ Osmolarity System, and clinical conjunctival inflammation. MMP-9 concentration and conjunctival inflammation were graded semi-quantitatively. Differences between anophthalmic sockets and the healthy fellow eyes for OSDI scores, MMP-9, TFO values, clinical conjunctival inflammation, and eyelid abnormalities as well as the correlation between these factors and demographic data were evaluated.

**Results:**

Patients had significantly higher OSDI, MMP-9, and TFO values, as well as higher conjunctival inflammation on the anophthalmic side, compared to the healthy side (*p* ≤ 0.002, respectively). For anophthalmic sockets, there was a significant positive correlation between OSDI scores and TFO values (*p* = 0.007), between the grade of posterior blepharitis and TFO values (*p* = 0.026), and between the conjunctival inflammation and MMP-9 values (*p* < 0.001), as well as between MMP-9 levels and time since eye loss (*p* = 0.004).

**Conclusions:**

Measuring MMP-9 and TFO may be helpful tools as efficient, quantifiable biomarkers, disease course parameters, or predictors for treatment response in the clinical management of patients with DASS or future therapy studies. Ophthalmologists should consider the updated diagnosis criteria including TFO and the definition for DASS proposed in this study.

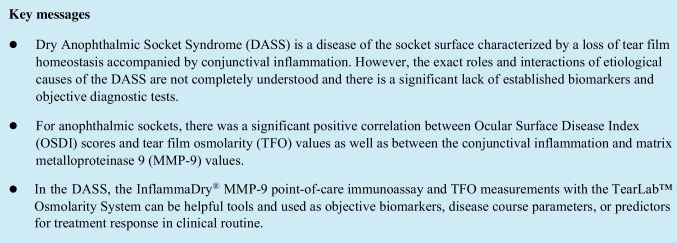

## Introduction

Dry anophthalmic socket syndrome (DASS) is a disease of the socket surface characterized by a loss of tear film homeostasis accompanied by socket discomfort, in which tear film instability, conjunctival inflammation, and damage, as well as eyelid and neurosensory abnormalities, play etiological roles [1, 2]. DASS affects most anophthalmic patients and is a significant cause of socket discomfort and reduced quality of life [1–19]. Previous studies have determined the following diagnostic criteria for DASS: the presence of subjective symptoms in the anophthalmic socket evaluated with standardized measurements (OSDI ≥ 13, SANDE ≥ 13, or DEQ-5 ≥ 6) and at least one of the five following clinical abnormalities—anterior blepharitis, posterior blepharitis, abnormalities of the meibomian glands (MGs) in the in vivo laser scanning confocal microscopy (LSCM), reduced tear meniscus height, and conjunctival inflammation [1, 2].

However, there is a significant lack of established biomarkers, diagnostic tests, and evidence-based treatment concepts for DASS [1, 2, 13]. The overall goal is to develop an evidence-based specific treatment algorithm for the DASS. Whether existing treatment concepts for dry eye disease can also be used or totally new treatment algorithms must be developed is unclear. But before developing treatment concepts, the exact roles and interactions of etiological causes of DASS have to be fully understood [1, 2, 13, 17]. In particular, the role of the tear film osmolarity (TFO) in DASS has not yet been investigated [1, 2, 13]. Changes in the TFO, especially hyperosmolarity, can lead to dry eye disease with related tear hyperosmolarity and disease severity [20–24]. Hyperosmolarity might therefore also play a significant role in DASS.

With the TearLab™ Osmolarity System (TearLab™ Corporation, USA) reproducible and accurate TFO measurements in a range between 270 and 400 mOsm/L and can be performed rapidly using small tear volumes of 50 nL [25, 26]. A point-of-care TFO test could be a beneficial instrument, as it may be used as an efficient, quantifiable biomarker, disease course parameter, and predictor for treatment response in clinical routine and future therapy studies in patients with DASS [25–27].

Conjunctival inflammation is a fundamental component of both dry eye disease and DASS [1, 2, 23, 24, 27–29]. Various inflammatory cytokines, released during the vicious cycle of ocular surface inflammation, have been identified [24, 27–31]. Cytokines such as matrix metalloproteinase 9 (MMP-9) play a crucial role in inflammatory pathways [24, 27–31]. Nowadays, an established MMP-9 point-of-care immunoassay (InflammaDry®) allows ophthalmologists to assess MMP-9 levels from tear film samples in a couple of minutes [27, 28, 32–34]. InflammaDry® is a simple and non-invasive, but accurate point-of-care assay identifying MMP-9 levels higher than 40 ng/mL [27, 28, 32–34]. The InflammaDry® MMP-9 point-of-care immunoassay might be a helpful tool in clinical routine and has the potential to be used as a quantifiable biomarker for socket inflammation in DASS.

The authors are not aware of any systematic prospective study evaluating TFO and MMP-9 levels in DASS or comparing TFO values and MMP-9 levels between anophthalmic sockets and healthy fellow eyes.

Therefore, the aims of this prospective study are to compare the TFO values and MMP-9 levels between anophthalmic sockets and fellow eyes and to assess the use of the MMP-9 point-of-care immunoassay and TFO measurements as biomarkers for the DASS.

## Patients and methods

Over 21 consecutive working days, patients who underwent ocular prosthetic care at the Trester-Institute for Ocular Prosthetics and Artificial Eyes, Cologne, Germany, were asked to participate in an extensive study regarding the use of biomarkers in the DASS. The study was approved by the Institutional Review Board of the University of Cologne (19–1277) and conducted independently by the Department of Ophthalmology, University of Cologne, Cologne, Germany, in adherence to the tenets of the Declaration of Helsinki and its later amendments. Informed consent was obtained from all participants after explanation of the study methodology. Inclusion criteria were adequate command of the German language and having worn a unilateral prosthetic eye made from cryolite glass for at least 1 year. Patients with a positive history of any ocular surface disease except dry eye disease caused by blepharitis, laser or surgical interventions, or contact lens wear on the healthy fellow eye were excluded. Also excluded were patients with topical use of anti-inflammatory drugs such as corticosteroids in the healthy fellow eye or at the anophthalmic socket in the last 6 months, the use of eye drops that could cause dry eye such as glaucoma drugs currently or in the past, and patients having socket or eyelid surgery in the last 3 months. In addition, patients with bilateral, defective, or poor-fitting prosthetic eyes were excluded as well as patients with a history of chemotherapy, systemic diseases causing dry eye, facial palsy, intravitreal operative injections, trigeminus or other facial nerve lesions, and any occlusion of the lacrimal system.

Firstly, patients were asked face-to-face to complete a questionnaire. This questionnaire was based on a previously developed, standardized questionnaire for the evaluation of the DASS. If the questions raised any issues during the questioning, they were answered directly. Data were gathered on age, gender, ethnicity, cause of eye loss, and date of eye loss as well as the date of fitting the present prosthesis, type of surgery, prosthesis cleaning frequency, and handwashing frequency before prosthesis removal. In addition, the history of topical medication in the anophthalmic socket and the healthy fellow eye at the current time point was queried. It was not noted whether or not the artificial tears contained benzalkonium chloride (BAK). The last section of the questionnaire included the German version of the ocular surface disease index (OSDI). Patients filled in this established and standardized questionnaire separately for the anophthalmic socket and the healthy fellow eye, always starting with the right side. All vision-related questions were classified as “not answered” for the anophthalmic side, similar to previous studies [1, 2]. The total OSDI score was then calculated based on the following formula as suggested and established in many previous studies: OSDI score = [(sum of scores for all questions answered) × 100] / [(total number of questions answered) × 4] [1, 2, 35].

Following this survey, palpebral conjunctival inflammation was graded analogous to Pine et al.’s 0–4 grading scale [36] with a ratio level of measurement, and the presence of lower eyelid abnormalities including ectropion, entropion, and lagophthalmos was evaluated, respectively. If an inversion or eversion of the lower eyelid was clinically visible, entropion and ectropion were nominally graded as present. The presence of a lagophthalmos was defined as the inability to close the eyelids completely upon request.

Any clinically visible inflammation of the eyelid skin, squamous debris, collarettes, or eyelash follicles was defined as anterior blepharitis, while dilated and telangiectatic lid margin blood vessels or plugging or displacement of the ductal openings were determined as posterior blepharitis. Anterior and posterior blepharitis were graded as absent (0), trace (1), mild (2), moderate (3), and severe (4) with a ratio level of measurement, respectively.

Afterward, tear film osmolarity (TFO) was measured in accordance with the manufacturer’s instructions using the TearLab™ Osmolarity System (TearLab™ Corporation, USA) for the anophthalmic socket and the healthy fellow eye, always beginning with the right side [25, 26]. The TearLab™ Osmolarity System was calibrated daily before the first measurement. The measurements were performed in a closed room under standardized light, temperature, and humidity conditions. A 50-nL tear sample film was obtained from the lateral canthus of the tear meniscus without touching the eye or the eye prosthesis. Osmolarity values were measured in mOsm/L. Results were graded as normal (≤ 300 mOsm/L), mild (301–320 mOsm/L), moderate (321–340 mOsm/L), and severe (≥ 341 mOsm/L) with a ratio level of measurement.

In addition, a matrix metalloproteinase 9 (MMP-9) point-of-care immunoassay (InflammaDry®, Quidel® Corporation, San Diego, USA) test was performed according to the manufacturer’s instructions, also beginning with the right side. The sampling fleece was dabbed at various locations along the inside of the patient’s palpebral conjunctiva of the lower eyelid, releasing the lid every 2 to 3 dabs to allow the patient to blink. After completing at least 6 to 8 dabs along the conjunctiva, the sampling fleece was rested against the conjunctiva for additional 5 s. Then, the test was assembled placing the sampling fleece of the sample collector into the sample transfer window of the test cassette body. Afterward, the sampling fleece was immersed in a buffer vial for a minimum of 20 s. After removing the sampling fleece from the buffer vial, the protective cap was replaced, and the test kit was placed on a flat surface horizontally. After 10 min, results were evaluated. If there was a streaky fluid wave in the background or if the test was negative after ten minutes, an additional 10 min was allowed to elapse before interpretation. All results without a blue line in the interpretation window were determined as invalid and therefore excluded from the study. Results with a blue line and any red line were considered positive, while results with a blue line but without a red line were determined as negative. Since the signal intensity of the test result increases proportionally to an increasing concentration of MMP-9 levels, the intensity of the red line was graded semi-quantitatively in all positive tests using an established grading index for a more detailed evaluation. The positive red line was compared with the grading index to classify the results as trace-positive, weak-positive, positive, and strongly positive with a ratio level of measurement.

### Statistical analyses

Commercial software (SPSS Version 26.0 for Mac; SPSS, Inc., Chicago, IL) was used for all statistical analyses. Shapiro–Wilk tests were performed to analyze the normal distribution of the blepharitis severity, OSDI scores, results of the MMP-9 point-of-care immunoassay, TFO values, and the conjunctival inflammation score. Due to the non-normal distribution, Wilcoxon tests were used to compare the severity of blepharitis, OSDI scores, MMP-9 and TFO values, and the conjunctival inflammation score between the anophthalmic socket and the healthy fellow eye.

Due to the non-normal distribution, Mann–Whitney *U* tests were used to compare the results of OSDI, the MMP-9 immunoassay, TFO values, and conjunctival inflammation score between enucleated and eviscerated anophthalmic sockets. Spearman’s rank-order correlation tests were used to investigate correlations between OSDI scores, results of the MMP-9 immunoassay, TFO values, and conjunctival inflammation score, respectively.

To investigate factors related to the TFO of the anophthalmic sockets, a linear regression model with the explanatory variables of age, gender (male vs. female), ethnicity (European or not), type of surgery (enucleation vs. evisceration), cause of eye loss (accident or not), years of wearing a prosthesis, wearing the prosthesis at night (yes or no), hand washing before prosthesis removal (yes or no), cleaning frequency (at least daily or less than daily), lower eyelid abnormalities including ectropion and entropion, and the severity of blepharitis anterior and posterior (absent, trace, mild, moderate, severe) was performed using backward elimination. To analyze factors related to the MMP-9 values of the anophthalmic sockets, a further linear regression model with the same explanatory variables as in the first model, but with two additional explanatory variables including TFO and conjunctival inflammation score, was performed also using backward elimination. Lastly, a third general linear model with backward elimination was performed to investigate factors associated with the OSDI scores using the same explanatory variables as in the second model, but also using the MMP-9 values as an additional explanatory variable. The threshold for statistical significance was set at *p* < 0.05. Furthermore, *R*^*2*^ and beta coefficients were calculated. *R*^2^ is a statistical measure of the fit of a regression model. *R*^2^ indicates how much variation in a dependent variable is explained by the independent variables. The values of *R*^2^ range from 0 to 1. *R*^2^ values < 0.3 indicate rather weak effects on the dependent variable, *R*^2^ values between 0.3 and 0.5 moderate effects, and *R*^2^ values > 0.7 strong effects. *R*^2^ = 1 means that a dependent variable is fully explained by the independent variables.

Beta coefficients are regression coefficients. Beta coefficients are standardized that allow a comparison of the magnitude of their effects directly. Possible values range from − 1 to + 1. A correlation coefficient of 0 suggests no correlation, while + 1 is indicating perfectly positive correlation and − 1 a perfectly negative correlation.

## Results

### Demographics of study population

Out of 204 patients who were approached to participate, 99 complied with the exclusion and inclusion criteria. Of these 99 patients, 1 patient declined to participate due to lack of time. Ninety-eight patients were finally enrolled in this study (Table 1).

**Table 1:** Demographics of 98 anophthalmic patients with at least one year experience in wearing cryolite glass prosthetic eyes

Characteristics of 98 study participants	
Gender	
Male, *n* (%)	61 (62.2%)
Female, *n* (%)	37 (37.8%)
Ethnicity	
European, *n* (%)	90 (91.8%)
Middle East, *n* (%)	6 (6.1%)
Asian, *n* (%)	2 (2.0%)
Age (years)	
Male, *mean* ± *SD*; *median* (*min–max*)	62.86 ± 16.69; 65.75 (23–89)
Female, *mean* ± *SD*; *median* (*min–max*)	61.85 ± 17.36; 62.75 (20–90)
Anophthalmic side	
Right, *n* (%)	57 (58.2%)
Left, *n* (%)	41 (41.8%)
Reason for eye loss	
Accident	54 (55.1%)
Medical	36 (36.7%)
Congenital	8 (8.2%)
Operation	
Enucleation, *n* (%)	85 (86.7%)
Evisceration, *n* (%)	13 (13.3%)
Mean time since current prosthesis fitted (years)	
Male, *mean* ± *SD*; *median* (*min–max*)	1.43 ± 1.20; 1.00 (0–7)
Female, *mean* ± *SD*; *median* (*min–max*)	1.24 ± 1.54; 1.00 (0–10)
Mean time since eye loss (i.e., time since surgery; years)	
Male, *mean* ± *SD*; *median* (*min–max*)	34.02 ± 23.83; 32.75 (1–79)
Female, *mean* ± *SD*; *median* (*min–max*)	34.47 ± 24.41; 31.08 (1–84)

*SD*, standard deviation; *min*, minimum; *max*, maximum

### Current topical medication and prosthesis care

Fourteen (14%) patients used artificial tears, and 15 (15%) used a nurturing eye ointment on the anophthalmic socket, while 14 (14%) used artificial tears, and one (1.0%) was currently using eye ointments in the healthy fellow eye. Seventy-seven patients (79%) cleaned their prosthesis at least once daily, ten (10.2%) less frequently than daily but up to and including weekly, two (2.0%) between weekly and monthly, and nine (9.2%) less frequently than monthly. Seventy-six (76) patients (78%) washed their hands always, 11 (11.2%) mostly, and 11 (11.2%) sometimes or never before removing and cleaning the prosthetic eye.

### Eyelid abnormalities

Of the 98 anophthalmic patients, nine (9%) had entropion, seven (7%) had ectropion, and 38 (39%) had lagophthalmos on the anophthalmic side. In contrast, on the fellow side, none had entropion, and 2 (2%) had ectropion. Fifty-nine (59) anophthalmic sockets (60%) had anterior blepharitis, with 32 (33%) having trace, 19 (19%) mild, seven (7%) moderate, and one (1%) severe disease. In addition, 58 (59%) had posterior blepharitis, with 29 (30%) having trace, 20 (20%) mild, seven (7%) moderate, and two (2%) severe disease. In contrast, out of the 98 fellow eyes, only 37 (38%) had anterior blepharitis, with 31 (32%) having trace and six (6%) mild disease. Furthermore, 37 (38%) had posterior blepharitis with 30 (31%) having trace and seven (7%) mild disease. None of the fellow eyes had any moderate or severe blepharitis. Patients had significantly higher anterior and posterior blepharitis on the anophthalmic side compared to the healthy eye (Wilcoxon tests, *p* < 0.001, respectively).

### OSDI, Pine’s conjunctival inflammation score, InflammaDry® matrix metalloproteinase 9 point-of-care immunoassay, and tear film osmolarity in anophthalmic sockets compared to the fellow eye

Patients had significantly higher OSDI scores on the anophthalmic side compared to the healthy side (Wilcoxon test, *p* < 0.001, Table 2). The mean OSDI score for the anophthalmic side was 17.67 ± 17.16 and for the fellow side 8.09 ± 10.84. Forty-six patients (47%) had a normal OSDI score on the anophthalmic side, while 22 (22%) had mild, 14 (14%) moderate, and 16 (16%) severe symptoms.

**Table 2:** OSDI, InflammaDry® point-of care MMP-9 immunoassay, tear film osmolarity, and Pine’s conjunctival inflammation score compared between the anophthalmic socket and the fellow eye, respectively

	All anophthalmic sockets (*n* = 98)	Sockets of enucleated eyes (*n* = 85)	Sockets of eviscerated eyes (*n* = 13)	Fellow eyes (*n* = 98)
OSDI, *mean* ± *SD*; *median* (*min–max*)	17.67 ± 17.16; 15.00 (0.0–75.0)	17.11 ± 16.53; 15.00 (0.0–75.0)	21.28 ± 21.27; 20.00 (0.0–60.0)	8.09 ± 10.84; 4.50 (0.0–58.3)
Normal (0 < 13), *n* (%)	46 (46.9%)	40 (47.1%)	6 (46.2%)	77 (78.6%)
Mild (≥ 13 < 23), *n* (%)	22 (22.4%)	19 (22.4%)	3 (23.1%)	14 (14.3%)
Moderate (≥ 23 < 33), *n* (%)	14 (14.3%)	14 (16.5%)	0 (0.0%)	4 (4.1%)
Severe (≥ 33), *n* (%)	16 (16.3%)	12 (14.1%)	4 (30.8%)	3 (3.1%)
InflammaDry® (MMP-9), *mean* ± *SD*; *median* (*min–max*)	1.74 ± 1.74; 1.00 (0.00–4.00)	1.79 ± 1.74; 1.00 (0.00–4.00)	1.46 ± 1.81; 0.00 (0.00–4.00)	0.04 ± 0.25; 0.00 (0.00–2.00)
Negative (0), *n* (%)	41 (41.8%)	34 (40.0%)	7 (53.8%)	95 (96.9%)
Trace positive (1), *n* (%)	12 (12.2%)	11 (12.9%)	1 (7.7%)	2 (2.0%)
Weak positive (2), *n* (%)	4 (4.1%)	4 (4.7%)	0 (0.0%)	1 (1.0%)
Positive (3), *n* (%)	13 (13.3%)	11 (12.9%)	2 (15.4%)	0 (0.0%)
Strong positive (4), *n* (%)	28 (28.6%)	25 (29.4%)	3 (23.1%)	0 (0.0%)
Tear film osmolarity, *mean* ± *SD*; *median* (*min–max*)	308.06 ± 23.41; 304.00 (275.00–380.00)	308.06 ± 23.41; 304.00 (275.00–380.00)	307.46 ± 33.102; 295.00 (275.00–370.00)	299.65 ± 16.20; 298.00 (275.00–386.00)
Normal (≤ 300 mOsm/L)	44 (44.9%)*	36 (42.4%)	8 (61.5%)	61 (62.2%)
Mild (301–320 mOsm/L)	30 (30.6%)	28 (32.9%)	2 (15.4%)	31 (31.6%)
Moderate (321–340 mOsm/L)	15 (15.3%)	15 (17.6%)	0 (0.0%)	3 (3.1%)
Severe (≥ 341 mOsm/L)	9 (9.2%)	6 (7.1%)	3 (23.1%)	3 (3.1%)
Pine’s inflammation score *mean* ± *SD*, *median* (*min–max*)	1.41 ± 1.03; 1.0 (0–4)	1.41 ± 1.08; 1.0 (0–4)	1.38 ± 0.65; 1.0 (0–2)	0.54 ± 0.69; 0.0 (0–2)
Absent (0), *n* (%)	20 (20.4%)	19 (22.4%)	1 (7.7%)	56 (57.1%)
Minimal (1), *n* (%)	36 (36.7%)	30 (35.3%)	6 (46.2%)	31 (31.6%)
Mild (2), *n* (%)	26 (26.5%)	20 (23.5%)	6 (46.2%)	11 (11.2%)
Moderate (3), *n* (%)	14 (14.3%)	14 (16.5%)	0 (0.0%)	0 (0.0%)
Severe (4), *n* (%)	2 (2.0%)	2 (2.4%)	0 (0.0%)	0 (0.0%)

^*^2 of these 44 patients had a tear film osmolarity ≤ 300 mOsm/L on the anophthalmic socket but had an inter-eye difference > 8 mOsm/L, indicating tear film instability and loss of homeostasis despite tear film osmolarity ≤ 300 mOsm/L at the anophthalmic side. *SD*, standard deviation; *min*, minimum; *max*, maximum

InflammaDry® MMP-9 point-of-care immunoassay mean value for the anophthalmic side was 1.74 ± 1.74 and for the fellow side 0.04 ± 0.25 with significantly higher values on the anophthalmic side compared to the healthy eye (Wilcoxon test, *p* < 0.001, Table 2). Forty-one patients (42%) had negative results, while 12 (12%) trace positive, 4 (4%) weakly positive, 13 (13%) positive, and 28 (29%) had strongly positive results on the anophthalmic side.

Analysis of the TFO also showed significantly higher values on the anophthalmic side compared to the healthy eye (Wilcoxon test, *p* = 0.002, Table 2) with a mean value of 308.06 ± 23.41 for the anophthalmic side and 299.65 ± 16.20 for the fellow side. While 54 (55%) had elevated TFO over 300 mOsm/L in the anophthalmic socket, 44 patients (45%) had normal values (≤ 300 mOsm/L). However, two of these 44 patients had a normal TFO in the anophthalmic socket, whereas an inter-eye difference of > 8 mOsm/L indicated tear film instability and loss of homeostasis on the anophthalmic side.

In addition, patients had significantly higher conjunctival inflammation on the anophthalmic side compared to the healthy eye (Wilcoxon test, *p* < 0.001, Table 2).

There were no significant differences between enucleated and eviscerated sockets for OSDI, InflammaDry® matrix MMP-9 immunoassay, TFO, and Pine’s conjunctival inflammation score (Mann–Whitney *U* tests, *p* ≥ 0.302, respectively).

### Associations between OSDI, Pine’s conjunctival inflammation score, InflammaDry® MMP-9 point-of-care immunoassay, and tear film osmolarity in anophthalmic sockets

There was a significant correlation between the OSDI score and values of TFO for anophthalmic sockets (Spearman’s rank-order correlation test, *p* = 0.006, Table 3). Furthermore, there was a significant positive correlation between Pine’s conjunctival inflammation score and the values of the MMP-9 immunoassay (Spearman’s rank-order correlation test, *p* < 0.001).

**Table 3:** Spearman’s rank-order correlation coefficients and *p* values between the OSDI scores, tear film osmolarity values, InflammaDry® MMP-9 immunoassay results, and Pine’s conjunctival inflammation score of the anophthalmic sockets, respectively

	OSDI	Tear film osmolarity	InflammaDry® MMP-9 immunoassay	Pine’s inflammation score
OSDI	-	0.278 (*p* = 0.006)	− 0.013 (*p* = 0.902)	0.117 (*p* = 0.251)
Tear film osmolarity	0.278 (*p* = 0.006)	-	− 0.141 (*p* = 0.165)	0.015 (*p* = 0.884)
InflammaDry® MMP-9 immunoassay	− 0.013 (*p* = 0.902)	− 0.141 (*p* = 0.165)	-	0.454 (*p* < 0.001)
Pine’s conjunctival inflammation score	0.117 (*p* = 0.251)	0.015 (*p* = 0.884)	0.454 (*p* < 0.001)	-

### Factors associated with tear film osmolarity, InflammaDry® MMP-9 point-of-care immunoassay, and OSDI in anophthalmic sockets

All linear regression models for TFO, MMP-9, and OSDI were statistically significant (analysis of variance: *p* = 0.002, *p* < 0.001, and *p* < 0.001, Table 4). In all three linear regression models the *R*^2^ values (*R*^2^ = 0.169, *R*^2^ = 0.276, and *R*^2^ = 0.257, respectively) indicated a rather weak influence of the independent variables on the dependent variable. The linear regression model for TFO suggests a significant correlation between the grade of posterior blepharitis and gender, as well as the cause of eye loss and TFO (*p* = 0.026, *p* = 0.026, and *p* = 0.008, respectively). The linear regression model for the values of the MMP-9 immunoassay confirms the significant association between the conjunctival inflammation score and MMP-9 immunoassay values from Spearman’s rank-order correlation test (*p* < 0.001), but also identifies a positive correlation between the MMP-9 levels and the duration of prosthesis wear, i.e., the time since eye loss (*p* = 0.004, Table 4). The third regression model suggests a significant correlation between gender, cause of eye loss, TFO, and the duration of prosthesis wear, i.e., the time since eye loss and OSDI values (*p* = 0.048, *p* = 0.001, *p* = 0.007, and *p* < 0.001, respectively).

**Table 4:** Association of explanatory variables with the values of tear film osmolarity, InflammaDry® MMP-9 immunoassay, and OSDI

Explanatory variables for tear film osmolarity (TFO)	Beta coefficient	95% confidence limits	*p*	
Gender (male [0] vs. female [1])	0.227	1.323–17.548	0.026	Female sex is associated with higher TFO
Cause of eye loss (accident [1] or not [0])	0.265	3.293–21.541	0.008	Accidental eye loss is associated with higher TFO
Severity of posterior blepharitis (0–4)	0.744	2.000–31.323	0.026	More severe posterior blepharitis is associated with higher TFO
Severity of anterior blepharitis (0–4)	− 0.597	− 29.544–1.287	0.072	-
Explanatory variables for InflammaDry® MMP-9 immunoassay	Beta coefficient	95% Confidence limits	*p*	
Years of wearing a prosthesis, i.e., times since surgery (years)	0.282	0.007–0.034	0.004	Longer time since eye loss is associated with higher MMP-9 values
Cause of eye loss (accident [1] or not [0])	− 0.186	− 1.301–0.004	0.051	-
Conjunctival inflammation score (0–4)	0.461	0.482–1.073	< 0.001	Higher conjunctival inflammation is associated with higher MMP-9 values
Explanatory variables for OSDI values	Beta coefficient	95% confidence limits	*p*	
Gender (male [0] vs. female [1])	0.194	0.049–13.614	0.048	Female sex is associated with higher OSDI values
Tear film osmolarity (mOsm/L)	0.259	0.052–0.328	0.007	Higher TFO vales are associated with higher OSDI values
Cause of eye loss (accident [1] or not [0])	0.351	5.010–19.110	0.001	Accidental eye loss is associated with higher OSDI values
Years of wearing a prosthesis (years)	− 0.351	− 0.389 to − 0.114	< 0.001	Longer time since eye loss is associated with lower OSDI values

*TFO*, tear film osmolarity; *MMP-9*, matrix metalloproteinase 9

## Discussion

Since the mean time since eye loss was more than 30 years, the majority of the study participants were very experienced and knowledgeable about dry socket complaints and anophthalmic socket inflammation. Although a limitation of the study was a single location, demographic data were very similar compared to previous studies and therefore represent the general anophthalmic population very well [1–8, 10–12, 15, 16, 37–39]. The results that there were no significant differences between enucleated and eviscerated sockets in patients with DASS and that patients in this study had significantly higher conjunctival inflammation on the anophthalmic side compared to the fellow eye were also in line with previous studies [1, 2, 5, 11, 16]. However, nearly 43% of the fellow eyes had minimal conjunctival inflammation and very mildly increased TFO values, mostly without any clinical symptoms, leading to the presumption that they have subclinical dry eye disease in the fellow eye.

Higher MMP-9 levels in patients with a longer duration of prosthesis wear, i.e., the time since eye loss, might be a consequence of chronic socket inflammation resulting in secondary morphological changes including atrophy of the meibomian glands [1]. The new finding that MMP-9 levels correlate with the grade of conjunctival inflammation is not surprising since the inflammatory cytokine MMP-9 has already been identified to play a crucial role in ocular surface inflammation and is established as a biomarker in dry eye disease [28, 32–34]. In fact, despite the highly prevalent condition of DASS in anophthalmic patients, diagnosis and clinical management remain problematic due to the lack of an evidence-based treatment protocol [1, 2]. Our results suggest that an MMP-9 point-of-care immunoassay (InflammaDry®) allows assessing MMP-9 levels very easily from small tear samples in a few minutes and might be used as a quantifiable biomarker for determining socket inflammation in DASS in clinical routine. Furthermore, the MMP-9 point-of-care immunoassay can be used for predicting treatment response and as a disease course parameter as well as a biomarker in future therapy studies helping to develop evidence-based treatment concepts for DASS.

In patients with increased MMP-9 levels, the use of artificial tears containing the preservative benzalkonium chloride (BAK) should be queried, since BAK can promote an inflammatory cycle and modify the level of MMP-9 [40]. This was also a significant limitation in this prospective study since the exact type of artificial tears was not noted. Therefore, anophthalmic patients should use preservation-free artificial tears to reduce inflammation and MMP-9 levels.

In unilateral anophthalmic patients, there are very different (anatomical) baseline situations between the anophthalmic socket and the fellow eye. To distinguish between the two sides/entities and to analyze unilateral disease, the OSDI must be asked for each side (the anophthalmic socket and the fellow eye) separately [1, 2, 35]. On the anophthalmic side, vision-related questions must be excluded [1, 2, 35]. The OSDI scores are to be calculated using the established formula depending on the number of answered questions separately for each side [1, 2, 35]. However, initially, the OSDI was designed to assess symptoms bilaterally for patients having two eyes [35, 41]. While the OSDI showed high specificity and sensitivity in patients with two eyes, specificity and sensitivity have not been fully investigated in anophthalmic patients [35, 41]. This could be a potential limitation of the study. Nevertheless, the methodology was successfully used in previous studies [1, 2]. The higher scores of OSDI for the anophthalmic socket compared to the fellow eye and the finding that most anophthalmic patients reported rather mild dry socket symptoms were in accordance with the results of previous studies [1, 2]. Most previous studies have not shown an absolute tear volume deficiency but rather a poor distribution of tears resulting in the absence of a sufficient tear film over the anterior surface which in turn could lead to dry socket complaints [1, 2]. Although more than 50% of all prosthetic eye wearers had at least mild dry socket symptoms, only 15% used artificial tears or ointments suggesting that dry socket symptoms might be accepted by anophthalmic patients as normal in the same way that they accept discharge to a certain degree or perhaps there is a lack of knowledge about DASS and/or of an evidence-based treatment concept [1–6, 11, 13, 15, 16]. In addition, despite more intensive care of the anophthalmic socket with eye ointments compared to the fellow eye, patients had significantly more symptoms, higher inflammation, and higher tear film osmolarity at the anophthalmic socket compared to the healthy side. This underlines our results.

Since there does not seem to be an absolute tear deficiency but rather a wrong distribution on the prosthesis surface, most previous studies have not shown a significant correlation between dry anophthalmic socket complaints and Schirmer test values [1, 2, 12]. Therefore, Schirmer tests were not performed in this study since they do not seem to provide sufficient diagnostic results in anophthalmic sockets [1, 2, 12]. However, the results of this study suggest that TFO changes, more precisely tear film hyperosmolarity, seem to play a crucial role in the etiology of DASS, and TFO changes seem to correlate with patients’ dry socket complaints. Due to these results, point-of-care TFO measurements with the TearLab™ Osmolarity System should be performed in daily practice instead of Schirmer tests in anophthalmic sockets. A reason for these TFO changes seems to be blepharitis posterior [42, 43]. Blepharitis posterior can lead to meibomian gland dysfunction resulting in a lipid layer deficit [42, 43]. This lipid layer deficit can lead to increased tear evaporation and consequently to a higher TFO [42, 43]. In addition, there was a significant positive correlation between the reason for eye loss and TFO as well as OSDI scores. Patients who lost their eye due to an accident (i.e., trauma) had significantly higher TFO and OSDI scores compared to those having (controlled) medical or congenital eye loss. The exact reasons stay unclear. However, traumatic eye loss resulting in various morphological changes including eyelid abnormalities, scarring, nerve damage, or irregularities of the ocular surface may be implicated in such difference.

The linear regression models also showed a significant positive correlation between gender, more precisely sex, and TFO as well as OSDI with females having significantly higher TFO and OSDI scores. Overall, sex, gender, and hormones play a major role in the regulation of ocular surface and adnexal tissues [44, 45]. These differences, similar to dry eye disease, might also play a significant role in the DASS [44, 45]. These factors and their impact on the DASS should also be investigated in further studies.

For a comprehensive evaluation, in addition to a detailed anamnesis and a questionnaire for dry anophthalmic socket complaints such as OSDI, ophthalmologists should evaluate patients using a standardized clinical examination. This examination should include a slit-lamp examination with regard to anterior and posterior blepharitis, eyelid position, blinking rate, and tear film break-up time [1, 2]. The fit and surface condition of the prosthesis should also be checked [1, 2]. Imaging of the meibomian glands, quantification of the tear meniscus and goblet cells, an examination of the lacrimal drainage system, and evaluation of the bacterial flora might also be useful [1, 2]. Since the use of Schirmer tests in anophthalmic sockets is not evidence-based, TFO measurements should be performed routinely, and MMP-9 point-of-care immunoassay (InflammaDry®) can be used additionally as a quantifiable biomarker for determining socket inflammation in DASS [1, 2, 12].

In summary, the DASS is a disease of the socket surface characterized by a loss of tear film homeostasis accompanied by socket discomfort, in which tear film instability, conjunctival inflammation, and damage, as well as eyelid and neurosensory abnormalities, play essential etiological roles [1, 2]. Based on the results of this study, the diagnostic set of DASS should be updated as follows: the presence of subjective symptoms in the anophthalmic socket evaluated with standardized measurements (OSDI ≥ 13, SANDE ≥ 13, or DEQ-5 ≥ 6) and at least one of the five following clinical abnormalities—anterior blepharitis, posterior blepharitis, tear film hyperosmolarity, abnormalities of MGs in the in vivo confocal LSCM, reduced tear meniscus height, clinical conjunctival socket inflammation resulting in conjunctival staining, or conjunctival inflammation determined with a MMP-9 immunoassay [1, 2]. Eye care practitioners should consider the updated diagnosis criteria including TFO when counseling anophthalmic patients. In the DASS, the InflammaDry® MMP-9 point-of-care immunoassay and TFO measurements with the TearLab™ Osmolarity System can be helpful tools and used as efficient, quantifiable biomarkers, disease course parameters, or predictors for treatment response in clinical routine. Furthermore, these biomarkers can be used in future therapy studies helping to develop evidence-based treatment concepts for DASS, which is a very high priority.
